# The impact of COVID-19 on chronic care according to providers: a qualitative study among primary care practices in Belgium

**DOI:** 10.1186/s12875-020-01326-3

**Published:** 2020-12-05

**Authors:** Katrien Danhieux, Veerle Buffel, Anthony Pairon, Asma Benkheil, Roy Remmen, Edwin Wouters, Josefien van Olmen

**Affiliations:** 1 University of Antwerp, Primary and Interdisciplinary Care, Doornstraat 331, 2610 Wilrijk, Belgium; 2 Institute of Tropical Medicine, Antwerp, Belgium; 3 University of Antwerp, Centre for Population, Family & Health, Antwerp, Belgium

**Keywords:** COVID-19, Chronic illness, Qualitative research, Continuity of care, Practice management, Telemedicine

## Abstract

**Background:**

The COVID-19 pandemic affects the processes of routine care for chronic patients. A better understanding helps to increase resilience of the health system and prepare adequately for next waves of the pandemic.

**Methods:**

A qualitative study was conducted in 16 primary care practices: 6 solo working, 4 monodisciplinary and 7 multidisciplinary. Twenty-one people (doctors, nurses, dieticians) were interviewed, using semi-structured video interviews. A thematic analysis was done using the domains of the Chronic Care Model (CCM).

**Results:**

Three themes emerged: changes in health care organization, risk stratification and self-management support. All participating practices reported drastic changes in organization with a collective shift towards COVID-19 care, and reduction of chronic care activities, less consultations, and staff responsible for self-management support put on hold. A transition to digital support did not occur. Few practitioners had a systematic approach to identify and contact high-risk patients for early follow-up. A practice with a pre-established structured team collaboration managed to continue most chronic care elements. Generally, practitioners expected no effects of the temporary disruption for patients, although they expressed concern about patients already poorly regulated.

**Conclusion:**

Our findings show a disruption of the delivery of chronic care in the Belgium prim care context. In such contexts, the establishment of the CCM can facilitate continuity of care in crisis times. Short term actions should be directed to facilitate identifying high-risk patients and to develop a practice organization plan to organize chronic care and use digital channels for support, especially to vulnerable patients, during next waves of the epidemic.

## Background

The COVID-19 pandemic has had a profound impact on public health, resulting in excess death rates of 170,000 in Europe and of more than 100,000 in the USA [1] during the first outbreak. However, not all people are equally affected by the virus; people with diabetes or cardiovascular disease had a 2,5–3,9 times higher odds of being infected [2], with infection generally resulting in worse outcomes and a higher mortality rate in elderly people and in patients with comorbidities such as hypertension, cardiovascular disease, chronic respiratory disease, chronic kidney disease and diabetes [3].

People with chronic conditions, however, are not only affected by the COVID-19 pandemic in a direct manner, but also in an indirect manner. The COVID-19 pandemic disrupts entire societies, including the routine health care systems. The unprecedented scale of this pandemic provided a significant challenge to modern medical care, requiring a collective shift towards the acute care for COVID-19 patients with severe presentation in hospitals, as well as the optimisation of infection control in the community. This comprehensive effort to contain the pandemic and minimize the subsequent morbidity and mortality has affected both the continuity and quality of care for patients with chronic diseases [4].

Resources at all levels have shifted away from chronic disease management and prevention during the outbreak, and the lock-down of many services has translated into reduced access, a decrease in referrals and reduced hospitalisations of patients with non-COVID-19 pathology [5]. Scattered reports suggest chronic patients have postponed health care seeking [6], some of them because of the fear of getting infected with the coronavirus [7]. In addition, patients have less options for community-based support and care. This leads to a serious concern about the indirect health footprint of COVID-19, especially on chronic diseases with increased complications and accelerated progression due to delayed and diminished access to secondary care and to a disruption in follow-up at primary care level.

These concerns indicate the need for an analysis of chronic care adaptation during the COVID-19 pandemic. Primary care providers have been struggling how to organise chronic care amidst the peak of the outbreak, when infection risk was high and resources extremely tight [8]. Pressure of COVID-19 on primary care is well documented, but the associated adaptation for chronic care is less so [9]. Chronic care models are based upon productive and active interactions between a patient, their informal care givers and the health care team, facilitated by a strong health care organisation and community embedding. Important research questions in relation to COVID-19 crisis are: How is such a model adapted in a context of a pandemic, in which the danger for serious and widespread infections absorbs most resources, and drastically changes the physical and social context in which to deliver care and support? How do primary care providers adapt their chronic care models to emerging crisis situations? How is the workforce adapted and what is prioritized? And what can we learn from these responses for the resilience of chronic care models?

This paper addresses this gap by examining the primary health care response among primary care providers in Belgium. The study aims to examine how both content and delivery of chronic care is being affected by the pandemic. Better understanding can enable us to identify ways to increase the resilience of the health system and be better prepared for flare-ups of the COVID-19 epidemic and other emergency situations.

## Methods

### Context and study population

This study was performed among primary care practices in Belgium. Belgium has registered an estimated 842,2 deaths per million inhabitants in the beginning of June [10], and the all-causes excess mortality of the first wave was the highest in Europe after Spain and England, almost double that of the USA. Five weeks after the first confirmed case in Belgium, in the context of a shortage of protection material, the federal government in collaboration with the National institute of Public Health: Sciensano issued an emergency plan for general practice with guidelines that stated to postpone all non-urgent care and to start triage centers [11] for COVID-19-suspected complaints. After emergent signals of an increasing burden on emergency units of non-COVID-19 patients with urgent problems having postponed needed care, the guidelines explicitly allowed the provision of chronic and psychiatric care [11] if urgent and to prevent worsening. Six weeks thereafter, general practices were allowed to re-open for non-urgent care, provided they maintained strict COVID-19-prevention and hygiene measures. With the introduction of the emergency plan, teleconsultations became permitted and remunerable, a novelty that had been postponed since many years. Suddenly, physicians were now expected to triage patients with complaints by phone. But teleconsultations were also explicitly allowed to guarantee the continuity of care for patients with chronic diseases. In this period 25% of Belgian patients saw their consultation with the general practitioner postponed, whereas in 33% of the cases it was switched to a teleconsultation [12].

The study was an additional study embedded in a larger study on the scale-up of integrated care for diabetes and hypertension, SCUBY [13]. General practitioners work traditionally as self-employed providers in small practices, but over recent eras some practices are being transformed into small multidisciplinary group practices. It concerns only a small number of practices, in which the GP stays the central health care professional, but delegates some tasks to e.g. nurses or dieticians. For the current study General practitioners (GPs) were recruited in a semi-rural area in the northern part of Belgium (Flanders), as in this region a mix of the different practices types was present. Practices were selected purposively, in order to reach different practice types and recruited by phone. Data collection occurred until data saturation, which was reached after twelve interviews. Purposive selection was done to recruit an equal number of GPs from different types of practices: solo working, monodisciplinary group practices and multidisciplinary group practices (with at least one nurse or a dietician).

### Data collection and analysis

Because of the COVID-19 related restrictions, interviews were held via a secured online video connection by 2 researchers. The interviews took place from over a 6 week period, starting in week 12 of the epidemic, 3 weeks after the initial peak. All interviews were recorded, transcribed verbatim and independently analyzed by the two first authors (KD and VB). They lasted 42 min on average. The interview guide contained questions about chronic care for diabetes and hypertension and an additional part with 12 questions about changes in care organization for patients with chronic diseases as a result from the COVID-19 (Additional file 1).

A thematic analysis was done using the elements of the Chronic Care Model (CCM) [14]: processes and incentives to improve the care delivery system; self-management support; team function and practice systems; evidence-based guidelines and implementation support; and information systems to facilitate the development of disease registries, tracking systems, and reminders and to give feedback on performance. The COREQ checklist was filled to assure complete reporting (Additional file 2).

## Results

Sixteen primary care practices were selected: 5 solo working, 4 monodisciplinary and 7 multidisciplinary. Within these practices, 21 people were interviewed: 3 dieticians (all female, 0 to 6 years of experience), 2 nurses (all female, 9 and 13 years of experience) and 16 GPs (9 female and 6 male, 1 to 42 years of experience). An overview of the participants is provided in Additional file 3. There were no important differences observed in the answers between male and female participants. Three major themes emerged: a) changes in health care organization; b) risk stratification; and c) self-management support.

### Changes in the health care delivery system and team approach

The providers all observed a general drop in consultations for chronic care. They mentioned the emergency plan and subsequent prohibition to see patient for non-urgent problems as the direct cause, but also referred to fear among patients.

*“Because they are afraid to come” (IV 17, GP, monodisciplinary group practice)*

In all primary care practices, the initial response was a re-organization with a focus on securing access to and safety of acute care with much attention to COVID-19 suspects. This entailed telephonic consultation and collaboration with triage posts for patients with COVID-19 suspected symptoms and the re-organization of the practice in line with the hygienic guidelines to enable access for patients with acute non-COVID-19 related health problems. This absorbed most time and energy, leaving little room to consider anything else, especially as defining what is urgent was not self-evident to the GP’s.

*‘In the beginning it was also very busy, so we just tried to do the most urgent. […] But what is urgent and what is not urgent? Chronic care becomes urgent also on a certain moment’ (IV 7, GP, multidisciplinary practice)*

The majority of primary care practices did not plan the (re-)organization of chronic care.

Nurses and dieticians were frequently put on temporary unemployment by the practice owner due to a loss of revenues following the drop in consultations and their services considered ‘not essential’. However, practices with an established culture of dialogue (such as a tendency to hold meetings regularly) took a more systemic approach with team meetings about organization and patients.

*‘Throughout the corona pandemic, so for seven weeks now, we have been meeting every afternoon for an hour about our patients, about the care, about the triage center, about having enough material, about cases, about yes, suicidal patients, about everything and more. Every day for an hour, so I think we are very alert for that and are fiercely engaged in doing the best possible care in this difficult period.’ (IV10, GP, multidisciplinary practice)*

Collaboration and concertation with medical specialists was more difficult for non-acute matters, also because not all referral centers communicated clearly about their changes in schedule and way of working. Access for acute care was no problem.

### Risk stratification and actively contacting patients

Few GPs had made a selection of high-risk patients to proactively contact to check whether they had medical or other problems. Most respondents recognized the value of such approach, but they mentioned barriers such as a lack of time and staff, ethical objections, and a limited knowledge on how to use the Electronic Medical Record (EMR) system.

*‘I have a problem with people calling patients myself. There are colleagues who do that, but I have a bit of a problem with that. I have a regular audience, they will come.’ (IV8, GP, solo practice)*

An important reported facilitator for pro-actively contacting patients was the availability of a list of the high-risk chronic patients extracted from the EMR, which was present in some larger group practices, and developed ad hoc in some others. GPs in solo practices indicated that they know their patients personally and that they would be able to identify high-risk patients by heart. When asked for examples of such patients, they mentioned different characteristics, such as those receiving home visits, of very old age, those not well-controlled, with recent change of medication, or patients reporting difficulties. GPs would approach such high-risk patients for a face-to-face consultation at their home or at the practice. Approaching patients actively was new to all GP’s.

*‘Firstly, we have coded everyone in our practice with chronic pathology: hypertension, diabetes, COPD, asthma. It has been very easy for us to draw lists. We also exported lists of patients with depression and oncological disorders. We started by calling the diabetes lists: if you get sick or if you feel anything contact us.’ (IV13, GP, multidisciplinary practice)*

In contrast to GPs, the dieticians interviewed stated the intention to contact all their clients for renewed appointments as soon as possible. This would also compensate for their unemployment during COVID-19.

### Self-management support

The new option of teleconsultation provided primary care practices with a potential tool to monitor and support patients with chronic diseases from a distance. However, most respondents said to mainly use these teleconsultations to prescribe medications and to get a quick overall impression of the patient.

*‘[...] actually, just verify how it's going. Are there any special complaints? Are they more tired? Can they still do their normal daily routine? Aren't they anxious with this corona virus?’ (IV3, GP, multidisciplinary practice)*

They had various reasons to resist to real, complete phone or video consultations. A frequently given answer related to unfamiliarity with this way of doing consultations and the perceived inability to assess patients well. Other arguments were the preference of patients, the lack of perceived need and the lack of time because of long-lasting COVID-19-related consultations.

*‘I cannot follow diabetes from a distance. I need to take lab tests, measure blood pressure.’ (IV8, GP, solo practice)*

*‘I did ask if they wanted it by phone or skype. But there are actually very few who have responded to that.’ (IV9, dietician, multidisciplinary practice)*

In addition, self-management support was usually provided by the nurse or dietician, but due to the lower revenues, these staff members were put on temporary unemployment.

*‘A nurse has not been able to work all the time, because everything a nurse does is not urgent or not essential or not life-threatening, or how should I put it.’ (IV10, GP, multidisciplinary practice)*

### Perceptions on the changes and effects on chronic patients

Respondents indicated that for the large majority of previously well-managed chronic patients the consequences of the COVID-19 outbreak and the associated re-organization of primary care would be limited. They argued that missing only one consultation is not problematic.

*‘Most of those who follow the quarterly check-ups and are stable, are not going to suddenly get worse.’ (IV21, GP, monodisciplinary group practice)*

However, there were worries about the effects on some patients, specifically those with socio-economic problems, whom they expected to experience more distress from COVID-19 and the lockdown. GPs mentioned that for these people, more unhealthy food and especially less physical exercise would probably be important causes of diabetes getting out of control.

*‘Because you know a lot of patients have had a lot less exercise than normal. They've only been able to find their salvation in the fridge. So in terms of pounds and exercise, that's been dramatic for a lot of patients in the last few weeks.’ (IV12, GP, monodisciplinary group practice)*

Most primary care practices were quite satisfied with the way their practice was organised and were proud of all the work they had done. Therefore, they did not plan on taking other measures next time, besides increasing their stock of protection material.

*‘I think that we as general practitioners and certainly we as a practice do that super well, and I think that from the side of the government some other things might have happened there.’ (IV10, GP, multidisciplinary practice)*

## Discussion

This study examined how primary care practices in a highly affected area in Belgium organized services for chronic conditions like diabetes and hypertension during the COVID-19 pandemic. Our findings show primary care providers themselves observed a severe disruption in the delivery of chronic care, which is in line with online surveys carried out during this period in Belgium [12, 15]. International research has proven this disruption had effect on multiple quality indicators [16, 17].

Despite the fact that Belgium is a high-income country which spends about 10% of its GDP on health care, previous studies [18–20] showed that patients with chronic conditions are often not receiving optimal care – also pre-COVID-19. These shortcomings permeate more strikingly in the primary care response to COVID-19. All the studied practices were forced to adapt to the crisis situation, but most practitioners did not deem a (re-)organization of chronic care necessary, relevant or possible. Some practices even missed their multidisciplinary colleagues or team members because of emerging financial constraints. This reduced the capacity of primary care practices even more to offer resilient chronic care and sheds light on the low priority given to self-management support, e.g. by paramedics.

It is thus obvious that a general re-organization of primary care for chronic conditions is needed – also to be able to adequately care for chronic conditions in times of a pandemic. A leading position paper clearly paved the way to reshape care for chronic conditions based upon the principles of Wagner’s Chronic Care Model (CCM) [21]. Our data seems to support this call for more comprehensive and integrated care: some larger and multidisciplinary teams that kept into contact with their chronic patients had established procedures for better care for their chronic patients and already deployed risk stratification and multidisciplinary collaboration. For example, one such practice (with a large multidisciplinary team and stable financing) managed to continue parts of chronic care, contacting vulnerable patients using online technologies. Our results suggest that the more successful adaption to the rapidly changing care environment was related to the pre-existing implementation of important domains of the chronic care model in the participating practices. Implementing the CCM in a practice may thus facilitate continuity of care in times of a pandemic.

We learned that most examined practices were not able to adjust quickly to changing circumstances. To reach the most vulnerable and frail chronic patients and to keep them as healthy as possible, risk stratification within a practice population is essential. The Kaiser Permanente’s triangle’ disease management model, focusing on intensive care and support of people with complex chronic conditions [14], may be used as an example. In the case of a new emergency, good record keeping and listing the patients in strata could help to keep in contact with these chronic patients. Pro-active measures may then be taken, using innovative techniques like telemedicine as a mitigation strategy as proposed by the World Health Organization. The potential of mobile and digital self-management support channels for patients can be further promoted especially among the pre-identified vulnerable patients [22].

The limitations or our study encompass the selection of our sample population and the lack of quantitative data of the care process and follow-up parameters. We sampled practices in one region in Belgium (Flanders), but a large variance of infection rates across the various regions exists as in the neighboring countries like the Netherlands. The experiences of the chronic patients were also beyond the scope of the present study. The strength of our study is that we interviewed different members of a range of primary care practices.

The COVID-19 pandemic and the associated challenges in ensuring quality chronic care have shed light on pre-existing deficiencies in primary care practices. Structural changes in primary care organization are needed to improve general chronic illness management as well as to be resilient to future COVID-19 waves and pandemics. Our qualitative study deepens the knowledge of how primary care practices vary in their organization of care in times of a pandemic and of chronic care in general. We suggest primary care practitioners to better identify the chronic patients in their patient population, and to proactively plan the steps to be taken in order to keep track of them, using a team-based approach. The study results generated two important pathways to achieve this (1): a more systematic implementation of the CCM, which could be achieved by better training of GP’s in practice management and (2) the establishment of a stable financing structure supporting staff (nurses and dieticians) so that they can play a role in managing chronic patients in times of a crisis. This will help to quickly switch between acute and chronic services and will improve continuity of care.

## Conclusions

This study shows that the COVID pandemic affected the continuity of chronic care drastically, casting light on pre-existing weak spots in chronic care organisation. Face-to-face consultations had to be ceased and focus shifted towards COVID care. In most practices there was no proactive reach out to patients with chronic diseases and multidisciplinary teamwork was pushed to the back burner. Otherwise, some good hope is present, as practices with reliable pre-existing structures did notably better. Important ways to improve are implementing the CCM through stratification of the patients according to their needs and planning ahead in anticipation of flare-ups or a second wave.

## Supplementary Information

**Additional file 1:** The impact of COVID-19 on chronic care_appendix1. Questionnaire used in the interviews.

**Additional file 2:** The impact of COVID-19 on chronic care_appendix2. COREQ checklist.

**Additional file 3:** The impact of COVID-19 on chronic care_appendix1. Overview of participants.

## Data Availability

The data that support the findings of this study are available on request from the corresponding author KD. The data are not publicly available due to them containing information that could compromise participant privacy.

## Abbreviations

CCM: Chronic care model
GP: General practitioner
EMR: Electronic medical record
COPD: Chronic obstructive pulmonary disease
GDP: Gross Domestic Product
