# Supplementary material for: The impact of COVID-19 on chronic care according to providers: a qualitative study among primary care practices in Belgium
Source: BMC Fam Pract. 2020 Dec 5;21:255. doi: 10.1186/s12875-020-01326-3 (PMC7718831; doi:10.1186/s12875-020-01326-3)
Supplement: Supplementary file 1 — Additional file 1: The impact of COVID-19 on chronic care_appendix1. Questionnaire used in the interviews. [file 12875_2020_1326_MOESM1_ESM.docx]

**Appendix 1. COVID-19 part of questionnaire**

1. How do you currently organize care for diabetes patients?
2. Which part of the patients still comes to consult?
3. Do you proactively contact some patients?
4. Which aspects are addressed in the telephone consultation?
5. Is there any coordination about the new way of working in your team? Do you consult with other team members on how to deal with it? Is there anyone who is in charge?
6. Can patients still get lab teste? How is the cooperation with the laboratory?
7. How is the cooperation with specialists for patients with chronic disease now?
8. As there are less community activities now, do you look for alternatives?
9. Do you now make more or less use of applications to support self-management?
10. Do you try to involve caregivers and family members more? Do they contact you more with questions?
11. What do you expect about the consequences of this pandemic and lock down on patients with chronic diseases? Do you think there will be groups who will suffer more or less?
12. If there would be a second wave in autumn, with a new lock down, how would you deal with it? Would you do things differently?
