# Supplementary material for: The impact of COVID-19 on chronic care according to providers: a qualitative study among primary care practices in Belgium
Source: BMC Fam Pract. 2020 Dec 5;21:255. doi: 10.1186/s12875-020-01326-3 (PMC7718831; doi:10.1186/s12875-020-01326-3)
Supplement: Supplementary file 2 — Additional file 2: The impact of COVID-19 on chronic care_appendix2. COREQ checklist. [file 12875_2020_1326_MOESM2_ESM.docx]

**Appendix 2. COREQ checklist**

| **No** | **Item** | **Guide questions/description** | **Answers** |
| --- | --- | --- | --- |
| **Domain 1: Research team and reflexivity** | | |  |
| Personal Characteristics | |  |  |
| 1. | Interviewer/facilitator | Which author/s conducted the interview or focus group? | See author's contributions and acknowledgements |
| 2. | Credentials | What were the researcher's credentials? *E.g. PhD, MD* | See acknowledgements and author's information |
| 3. | Occupation | What was their occupation at the time of the study? | See acknowledgements and author's information |
| 4. | Gender | Was the researcher male or female? | See acknowledgements and author's information |
| 5. | Experience and training | What experience or training did the researcher have? | KD and VB: trained and experienced in qualitative research. JA and TC: trained on the job by KD and VB |
| Relationship with participants | |  |  |
| 6. | Relationship established | Was a relationship established prior to study commencement? | No, participants recruited for this study in particular |
| 7. | Participant knowledge of the interviewer | What did the participants know about the researcher?  e*.g. personal goals, reasons for doing the research* | Info brochure stating the goal of the research and short introduction by the researchers |
| 8. | Interviewer characteristics | What characteristics were reported about the interviewer/ facilitator? e.g. *Bias, assumptions, reasons and interests in the research topic* | Info brochure stating the goal of the research and short introduction by the researchers |
| **Domain 2: study design** | |  |  |
| Theoretical framework | |  |  |
| 9. | Methodological orientation and Theory | What methodological orientation was stated to underpin the study? *e.g. grounded theory, discourse analysis, ethnography, phenomenology, content analysis* | Content analysis |
| Participant selection | |  |  |
| 10. | Sampling | How were participants selected?  *e.g. purposive, convenience, consecutive, snowball* | See methods, context and study population |
| 11. | Method of approach | How were participants approached?  e*.g. face-to-face, telephone, mail, email* | Telephone |
| 12. | Sample size | How many participants were in the study? | See results, first paragraph and appendix 2 |
| 13. | Non-participation | How many people refused to participate or dropped out? Reasons? | 34 participants refused, they were busy or not interested. This number is similar to other studies trying to sample GP’s in Belgium |
| Setting | |  |  |
| 14. | Setting of data collection | Where was the data collected? e*.g. home, clinic, workplace* | See methods, data collection and analysis |
| 15. | Presence of non-participants | Was anyone else present besides the participants and researchers? | No one present |
| 16. | Description of sample | What are the important characteristics of the sample?  *e.g. demographic data, date* | See appendix 2 |
| Data collection | |  |  |
| 17. | Interview guide | Were questions, prompts, guides provided by the authors?  Was it pilot tested? | See appendix 1 and author's contributions |
| 18. | Repeat interviews | Were repeat interviews carried out? If yes, how many? | No repeat interviews |
| 19. | Audio/visual recording | Did the research use audio or visual recording to collect the data? | See methods, data collection and analysis |
| 20. | Field notes | Were field notes made during and/or after the interview or focus group? | No field notes made |
| 21. | Duration | What was the duration of the interviews or focus group? | 42 minutes average |
| 22. | Data saturation | Was data saturation discussed? | See methods, context and study population |
| 23. | Transcripts returned | Were transcripts returned to participants for comment and/or correction? | No transcripts returned |
| **Domain 3: analysis and findings** | |  |  |
| Data analysis | |  |  |
| 24. | Number of data coders | How many data coders coded the data? | See methods, data collection and analysis |
| 25. | Description of the coding tree | Did authors provide a description of the coding tree? | No code tree |
| 26. | Derivation of themes | Were themes identified in advance or derived from the data? | Derived from the data |
| 27. | Software | What software, if applicable, was used to manage the data? | Microsoft Office Word and Excel |
| 28. | Participant checking | Did participants provide feedback on the findings? | No participant checking |
| Reporting | |  |  |
| 29. | Quotations presented | Were participant quotations presented to illustrate the themes / findings? Was each quotation identified?  e*.g. participant number* | See results |
| 30. | Data and findings consistent | Was there consistency between the data presented and the findings? | See results |
| 31. | Clarity of major themes | Were major themes clearly presented in the findings? | See results |
| 32. | Clarity of minor themes | Is there a description of diverse cases or discussion of minor themes? | See results |
