# Supplementary material for: The impact of COVID-19 on chronic care according to providers: a qualitative study among primary care practices in Belgium
Source: BMC Fam Pract. 2020 Dec 5;21:255. doi: 10.1186/s12875-020-01326-3 (PMC7718831; doi:10.1186/s12875-020-01326-3)
Supplement: Supplementary file 3 — Additional file 3: The impact of COVID-19 on chronic care_appendix1. Overview of participants. [file 12875_2020_1326_MOESM3_ESM.docx]

**Appendix 3. Overview of participants, characteristics and time of interview**

| Participant | Health profession | Interview date | Sex | Practice type |
| --- | --- | --- | --- | --- |
| 1 | GP | 2020-04-24 | Male | Group, mono |
| 2 | GP | 2020-04-24 | Male | Solo |
| 3 | GP | 2020-04-28 | Female | Group, multi |
| 4 | GP | 2020-04-29 | Female | Solo |
| 5 | GP | 2020-04-29 | Female | Solo |
| 6 | Dietician | 2020-04-30 | Female | Group, multi |
| 7 | GP | 2020-04-30 | Female | Group, multi |
| 8 | GP | 2020-04-30 | Male | Solo |
| 9 | Dietician | 2020-05-04 | Female | Group, multi |
| 10 | GP | 2020-05-05 | Female | Group, multi |
| 11 | Nurse | 2020-05-05 | Female | Group, multi |
| 12 | GP | 2020-05-06 | Male | Group, mono |
| 13 | GP | 2020-05-07 | Male | Group, multi |
| 14 | Dietician | 2020-05-08 | Female | Group, multi |
| 15 | GP | 2020-05-08 | Female | Group, multi |
| 16 | GP | 2020-05-08 | Female | Group, mono |
| 17 | GP | 2020-05-12 | Female | Group, mono |
| 18 | GP | 2020-05-12 | Male | Group, multi |
| 19 | Nurse | 2020-05-13 | Female | Group, multi |
| 20 | GP | 2020-06-05 | Female | Solo |
| 21 | GP | 2020-06-07 | Male | Group, mono |
